# Causation of Disease—Epidemics

**Published:** 1872-04

**Authors:** Frank A. Ramsey

**Affiliations:** Vice-President Tennessee Medical Society; President East Tennessee Medical Society; Corresponding Member Gynœcological Society, Boston; Resident Secretary of the Anthropological Society, London, etc., etc.


ATLANTA

### MEDICAL AND SURGICAL JOURNAL.

VOL. X.—APRIL, 1872.—No. 1.

### ORIGINAL COMMUNICATIONS.

### CAUSATION OF DISEASE—EPIDEMICS.

BY FRANK A. RAMSEY, M.D.,

*Vice-President Tennessee Medical Society; President East Tennessee Medical Society; Corresponding Member Gynæcological Society, Boston; Resident Secretary of the Anthropological Society, London, etc., etc.*

[Communicated by SWAN M. BURNETT, M.D., Secretary Medical Society East Tennessee.]

"A general statement of any system of philosophy may be either a sketch of a doctrine to be established, or a summary of a doctrine already established."—COMTE.

In the language of the medical profession, there occurs two words that are identical in etymological force, except, perhaps, a shadow of difference in the proportions of which they in part consist. Epidemic and endemic, when literally rendered, mean, each, upon the people and on the people. Conventionally, they are of very different import. The word endemic has a significance which is commonly accepted, and it is seldom, if ever, employed in such a manner as to give occasion to any misunderstanding or doubt of the idea in the mind of the person by whom it is employed, or as to produce any confusion in the mind of any one in considering the subjects in connection with which the word is used. Originally, when the two words were used, each had a distinctive force; they were antithetical. One has preserved its original conventional meaning; but, for a long time, the other has been employed in a manner that perverts it from the purpose to which it was designed, and obscures consideration of the circumstances intended to be comprehended in the word.

Sydenham, in conveying, during the seventeenth century, ideas that have been confirmed by all observation since, used the term epidemic with the design, doubtless, that it should be restricted to its antithetical position; but unfortunately, in illustrating, he invaded the precincts in meaning of the term endemic; and hence has resulted some difficulty in understanding the matter as he did, and his teachings have not, perhaps, maintained apodictical influence.

Though it is evident that medical writers, since Syndenham, almost as a totality, entertain the idea attached to the term in its antithetical position, they have, nevertheless, introduced it in such connections as to perpetuate difficulty in understanding the subjects that involved the use of the term. True, a few who have presumed to ignore Sydenham's teachings, and who have boldly denied truths of observation, have found it necessary to deepen the erroneous though somewhat extensively received meaning of the term, through which points, that are indeed settled, are kept seemingly under discussion. Thus Dr. Byrne, in his publication in 1843, and republished in 1855, says:—"The word epidemic signifies literally upon the people, and should be made to apply to all such diseases as prevail extensively among the people, whether they have their origin in the air, from the earth, or in the human body." And recognizing its original antithetical character, he appreciates the difficulty inherent in employing the word in the sense that he gives, and declares—"The term endemic I shall totally discard, as being calculated to cause confusion." But it is when employing "the speculations of reason, in and of themselves of paramount greatness and transcendency, is what we do *not* require," that words are torn from the places to which they belong, and degraded to obsolescence, and others trimmed to give speciousness to partial views of a matter under discussion—not when honestly regarding facts as they have been observed, and calmly attempting to place them in systematized form on the record.

By the permission of those who had themselves clear views of the essential meaning of epidemic, and hence, probably, did not feel sensitively the importance of restricting its use, the lesser number of writers have accomplished their purpose, and the term, at the present day, can not always be employed in its original conventional force without a qualifying word, or paragraphs in explanation. Every such qualitative becomes a new source of difficulty, and increases the already too great opportunity for discussion.

No better illustration of these affirmations could be desired than is furnished in the volumes of Transactions of the American Medical Association. Dr. Joseph Smith, of New-York, as Chairman, made the first report of the Committee on Practical Medicine, at the session held at Baltimore, in the year 1848. In its preparation, he had evidently a correct understanding of the essential character appertaining to an epidemic, but was constrained, under the very common employment of the word, to bring forward his own correctness of understanding in the premises, with the term weighted by another word, thus:—"*Meteoratus epidemics*—those wide-spreading maladies which have no special relations or connections with seasons, localities and climates." And the effect is noticeable. Subsequent reports, though afterwards made by a special committee on epidemics, seldom embrace anything on diseases really epidemic, but are abundantly full on the wider spread or less general prevalence than ordinary of diseases expected regularly at particular seasons and places, and of diseases from accidental causes, themselves produced by casual concurrence of circumstances within a defined area.

The whole medical profession is in this manner involved in the absurd category of attempting to improve upon, or ignoring, word-rendering by Noah Webster—to whose astuteness as a philologist, the scholars of America and Europe bear testimony, and to whose correctness in defining, the highest legal tribunal of Great Britain gives deference. The great lexicographer says: "Epidemic—a disease *generally* present, but not dependent upon any local morbific cause, and not confined to any season, climate, region, or country. It is used in distinction from endemic." Such, indeed, is the only meaning the term ought to have now, if it is to be retained and used at all—expressive of a class of diseases that must necessarily have a designative by which to be recognized without constant explanation, and to which purpose it is, in the existing state of medical records, better adapted than any other word.

The word pandemic has been employed to a limited extent, and inasmuch as its literal meaning is "all people," perhaps it would have been better had writers, in referring to certain diseases, and certain peculiarities in connection with diseases, availed themselves of its etymological force. But the etymological sense of words is soon lost—they can not retain it and continue in use; "and if we do not attach a conventional meaning to words, they must cease to be intelligible symbols."

Since the fourth century, when the conventional sense of the term epidemic was formally recorded by Ægeneta, it has always, when necessary, been used in that sense; and now, in the nineteenth century, it is declared by Webster, the highest authority, to bear the same meaning—to have the same conventional force. In the language of science, no word or form of expression ought to be permitted use in more than one sense; it ought to have but a single meaning. Hence, it is altogether improper to invade, as in this instance, the authority of old and continued use, and appropriate, as expressing many people in a limited area, a word that in truth expresses circumstances having relation to all habited surface, without regard to numbers of persons.

Epidemic diseases are those "whose generation being universal, their cause is so likewise." They are "engendered through occult and inexplicable" occurrences; they are dependent upon and proceed from conditions of which we are utterly ignorant—certain hidden and inexplicable modes in nature that occur here, there, and everywhere, and under any and every cognizable condition, always alike, each after its kind; "they prevail under every possible diversity of circumstance and situation, not only independently of, but actually bidding defiance to, all known contingencies." The hidden, inexplicable modes prevailing, the work begins, and consequent effects are not and can not be confined within prescribed limits of territory, or impeded by natural or artificial barriers, or modified by wet or dry seasons, or affected by temperature. The work accomplishes destruction without, probably, any assistance from the condition of the poorly-fed and badly-clothed, the dirty and dissolute, who furnish a large quota of victims, and probably without any leniency because of prudence, proper diet, cleanly habits, and security against the vicissitudes of weather, and in the strife in life, practiced and enjoyed by the well-to-do and the wealthy, who, too, furnish a large quota of victims. Or the hidden and inexplicable modes prevailing may not operate to the production of effects so distinctive as to receive a name, and be given position in a system of nosology; but seizing upon one or the other of the contagious diseases, or upon the disease of each locality and of each country, makes it the witness of the presence of an occult influence—constituting a mysterious cause—occasioning a wider spread than usual of the disease—increasing or diminishing some one or more of its ordinary features, or making all more or less intense—promoting, discouraging, changing, or instituting intercurrent affections, and lessening or making larger the relative mortality; thus forcing acknowledgment, as of an established fact, of the doctrine of epidemic constitution.

This may be expressed after another form. The contagions of measles, scarlet fever and small-pox, presumptively, are constantly in operation at some one or another point; but at different times, either one spreads as from a focus, and so suddenly embraces in its active exhibitions a large area of an inhabited country, and a large number of persons, as hardly to admit of explanation by the ordinary manner of communication. But grant that no one is attacked except from contact with the contagion, it is evident, at such times, that an influence wholly beyond any circumstances with which we are cognizant has given an energy to the poison that usually it does not have, or has aroused a susceptibility in persons which does not ordinarily prevail among the people. This occurrence of a larger area of country being visited, and a larger number of persons being impressed by the particular contagion, during the same season or the same year, in the absence of a rationale, is made satisfactory to the understanding that receives the doctrine of the uniform existence of an epidemic constitution—rubeolous, scarlatinal, or variolous—as the fact may be evidenced in the extensive prevalence of the one or the other disease at the same time in countries of different climates, or widely separated and differently circumstanced localities of the same country.

Particular regions of countries are regularly visited at the same season of the year with periodic fevers. Each visitation has its own peculiar features—differing one season from the features of the same kind of fever occurring during the like seasons of the previous year, and so uniformly observed as to be characteristic. Now, constipation requires the administration of purgative medicaments; but last year, laxness of the bowels prevented the employment of agents, whether as medicine or food, known to increase peristaltic action, or believed to invite the fluids to the mucous surface of the intestines, or render more active the secrements and exhalents of the first passages. Last year, the stomach was undisturbed in fever cases, and did not readily, under any kind of ingesta, give evidence of any disarrangement; but now, pain, nausea, readily-induced vomiting, are present in the many instances of fever. Now, headache, suffused eyes, injected conjunctiva, contracted pupils, and various forms of delirium, occur in those who are sick with fever; but last year, the intelligence remained natural, the eyes were not involved, the head was unaffected. Last year, patients with fever had bronchitis and pneumonia; now, the viscera of the chest preserve the state and condition in which they were when the individual was taken with fever. Now, bleeding is inadmissible; last year, persons died unless the lancet prevented. Last year, the relative number of persons attacked was very large; now, the relative number attacked is very small. Now, the relative mortality is large; last year, the relative mortality was small. During the same season, in other sections and other countries, continued fevers occur regularly; and there, precisely the same peculiarities, the same marked characteristics observed at localities where the periodic fevers are prevailing, are observed. The uniform, universal observation of the peculiarities, the characteristics, has not yet been successfully subjected to a rationale, and is made satisfactory only to the understanding that admits the doctrine of an existing epidemic constitution.

It occurs that some years disease of all kinds is less extensive in prevalence; but a few persons are attacked, and the disease in progress has but little intenseness, and there are but few deaths of the few persons who are attacked.

It occurs that some years disease of all kinds, without any ascendancy of a particular form, is very extensive in prevalence; very large numbers of persons are attacked, and the exhibitions of disease in progress are very intense, and the proportion of deaths to the number of the sick is very large.

These features are observed alike in Europe, Asia, Africa, and America—on the continents and the islands, on the mountains and in the valleys, on the sandy plain, and on ships in mid-ocean; everywhere there is relative salubrity, and where disease does present, it presents relatively with mildness, or everywhere disease is rife, severe, and results seriously—a uniformity that can not be explained except upon the hypothesis of hidden, inexplicable modes in nature effecting an epidemic constitution. This is the teaching of Sydenham:—"There are different constitutions in different years. They originate neither in their heat nor their cold, their wet nor their drought; but they depend upon certain hidden and inexplicable changes in nature occasioning the cause by which the bodies of men are predisposed and determined, as the case may be, to this or that complaint. This continues during the influence of this or that constitution, which gives ground and makes way for another." And hence a common saying, "All diseases wear the livery of the epidemic." This must be, necessarily, because, "the generation of the cause being universal, the effects must be so likewise." The epidemic constitution will be exhibited through known contagious diseases, and through diseases easily accounted for upon considering the circumstances of the place of their occurrence, and through diseases of accidental causation, and through diseases that belong to season. But sometimes the hidden and inexplicable modes in nature will be exhibited through a new assemblage of symptoms—or, as is true for the most part, through an assemblage of symptoms that is known to occur from accidental or permanent causes at particular places and during certain seasons, and occasion the assemblage to present in so large a number of persons, in so many various climates and during so great a variety of seasons, and with marked distinctness in severity, and preserving everywhere, under every difference of cognizable circumstances, an approach to the same ratio of mortality, that it ceases to be the disease that the practitioner of medicine encounters every year, and becomes an epidemic disease. It is the universality of its prevalence that gives it the character of epidemic, which universality of prevalence is not amenable to any explanation involving the consideration of circumstances at present within the knowledge of professional observation or scientific investigation. And universality of prevalence does not comprehend the play of the modes in nature everywhere at the same time; the modes are hidden and inexplicable, and when and where they will, and why they do exhibit action productive of dire effects, can not now be told. "It is not, indeed, unbecoming a philosopher, when he is ignorant, to acknowledge his ignorance, if he has used all diligence in searching for the truth; but it is highly unbecoming to pass off the unknown for the known, and the doubtful for the certain."

It has been very many years since Sir John Pringle remarked that "attempts to ascertain the cause of epidemics are, for the most part, more specious than substantial;" yet attempts have been constantly, and continue to be made, and of course still maintain the character of speciousness without substantiality. In every instance, the cause has been sought after as if generated or created in a locality, and starting from that locality, has, with greater or less rapidity, diffused itself hither and thither; and because of the assumption, always, that the cause is formed only at the locality at which the disease is first observed, and afterward recognized as epidemical, the manner of search after the essential nature of epidemical disorders, so far as connected with their cause, has been confined to the channels of human intercourse, and has been prosecuted in such wise as to have engendered partisan discussion upon incidental, intercurring, or side issues—distracted observation—perverted the application of facts—forced expenditure of the power of intellect upon creations of the imagination, and in effort to sustain or destroy gratuitous positions, and effectually prevented any, even the least, approach to the still hidden and inexplicable modes in nature that are requisite to the occurrence of the epidemic disease. The mind of man has all the while gone adrift from considering the cause as existing just where the disease prevails, and as having origin just where it exists, as embraced in the comprehensive definition of Ægeneta, making the generation of the cause as universal as the prevalence of the epidemical disease. In this sense, the disease may occur, as it has occurred, at low and high altitudes—in old and new countries—in sparsely-inhabited settlements and densely-crowded towns and cities—in mid-desert and mid-ocean—in summer and winter—and attack one or many, and prevail for but a few hours, and for many weeks, just where and when the hidden and inexplicable modes in nature occur, and just so long as the modes are maintained, and susceptible human economies are found to be impressed at the particular locality.

The reception of the definition of Ægeneta at least simplyfies the otherwise profound or multifidous mystery of the cause and movement, and destroys the otherwise inconsistencies that occur in any description of an epidemical disease.

The marked features of an epidemic are,—

1. Simultaneous, or consentaneous, at different and distantly-separated places.

2. Appearance and prevalence in a country and in towns, contiguous country and intercommunicating towns perfectly exempt.

3. The sudden, perhaps gradual, disappearance of the disease from the places at which it had prevailed; and subsequently—sometimes after a short, sometimes after a long interval—its sudden, perhaps gradual, appearance in the contiguous country and intervening towns which before were exempted, with or without reappearance at the parts of country and places of its previous occurrence.

4. Prevailing in one part of a town and leaving other parts in health — even confining its operation to one side of a narrow street, leaving the other without trouble; or operating on one side of a narrow river, and not on the other, notwithstanding the intermingling of atmospheres and constant communication between the inhabitants, and the sameness of habits, surrounding and cognizable conditions; or prevailing with one wing of an army, leaving the other untouched.

5. Appearance in an army at a locality, and prompt and entire disappearance so soon as the place of encampment is changed.

6. Appearance at any and every season of the year, in all quarters of the earth, and unaffected by hot or cold, wind or calm, wet or dry.

7. The disease claims its victims from all classes and conditions of society in the places of its occurrence.

8. Quarantine, military cordons and hygienic measures are ineffectual in preventing its appearance, or modifying the extent of prevalence in a place, or mollifying the disease in its progress in individuals.

